# Synthetic biology tools for engineering *Yarrowia lipolytica*

**DOI:** 10.1016/j.biotechadv.2018.10.004

**Published:** 2018-12

**Authors:** M. Larroude, T. Rossignol, J.-M. Nicaud, R. Ledesma-Amaro

**Affiliations:** a Micalis Institute, INRA, AgroParisTech, Université Paris-Saclay, 78350 Jouy-en-Josas, France; b Department of Bioengineering and Imperial College Centre for Synthetic Biology, Imperial College London, London, United Kingdom

**Keywords:** *Yarrowia lipolytica*, Synthetic biology, DNA assembly, CRISPR-Cas9, Genome editing, Golden Gate, Industrial biotechnology, Genome scale metabolic models, Metabolic engineering

## Abstract

The non-conventional oleaginous yeast *Yarrowia lipolytica* shows great industrial promise. It naturally produces certain compounds of interest but can also artificially generate non-native metabolites, thanks to an engineering process made possible by the significant expansion of a dedicated genetic toolbox. In this review, we present recently developed synthetic biology tools that facilitate the manipulation of *Y. lipolytica*, including 1) DNA assembly techniques, 2) DNA parts for constructing expression cassettes, 3) genome-editing techniques, and 4) computational tools.

## Highlights

• Due to its metabolic features, *Y. lipolytica* is a promising cell factory for producing compounds of biotechnological interest.
• Fast and efficient synthetic biology tools have been developed, which has boosted engineering strategies for *Y. lipolytica*.
• This review describes the latest advances in synthetic biology for *Y. lipolytica*.
• DNA assembly tools, DNA parts for expression cassettes, genome-editing techniques, and computational tools are covered here.

## 1 Introduction

*Yarrowia lipolytica* is a non-conventional dimorphic yeast with the potential to act as a biotechnological workhorse in a wide range of applications. This organism is often found in fermented foods such as cheese and meat, and it is a good natural producer of certain compounds of industrial interest, including citric acid, erythritol, and various proteins and lipids. The ability of *Y. lipolytica* to grow at high cell densities and to produce large titers of valuable molecules has attracted the attention of the scientific community. Consequently, research carried out with this organism is growing exponentially, which is clearly reflected in the increasing number of related articles and patents. Thanks to the yeast's characteristics and to the development of molecular biology tools specific to it, *Y. lipolytica* has been extensively engineered to produce chemicals and fuels (Cavallo et al., 2017; Guo et al., 2016; Ledesma-Amaro et al., 2016b; Ledesma-Amaro and Nicaud, 2016b).

Synthetic biology is an emerging discipline that aims to apply engineering principles to biological systems to render them more controllable, standardized, and predictable. These latter benefits can significantly accelerate research since new and highly efficient tools can be developed, allowing systems of interest to be constructed more rapidly and at a lower cost. In recent years, different synthetic biology tools have been generated and applied in *Y. lipolytica*, which has further expanded the range of applications for this yeast. *Y. lipolytica* has a metabolism that is well suited to fatty acid production and lipid accumulation and has consequently been used as a host organism for generating large amounts of lipids (Darvishi et al., 2017; Friedlander et al., 2016; Park et al., 2018a, Park et al., 2018b). More specifically, strains have been engineered to produce large amounts of oleic acid oil with no polyunsaturated fatty acid (Tsakraklides et al., 2018); unusual fatty acids such as hydroxy fatty acids and ricinoleic acid (Beopoulos et al., 2014); oils that resemble cocoa butter, which is rich in stearic acid and could thus be used as an oil substitute in chocolate production (Papanikolaou and Aggelis, 2003a); conjugated fatty acids such as conjugated linoleic acids (Imatoukene et al., 2017; Zhang et al., 2013, Zhang et al., 2012); and omega-6 and omega-3 fatty acids like EPA (eicosapentaenoic acid, C20:5) (Xue et al., 2013). The latter process has led to two commercial products by DuPont (Xie et al., 2015).

Other valuable compounds that have been produced include methyl ketones (Hanko et al., 2018); polyhydroxyalkanoates (PHAs), which are good candidates for use in renewable and biodegradable bioplastics (Gao et al., 2015; Haddouche et al., 2011, Haddouche et al., 2010; Li et al., 2017); carotenoids (Kjaergaard et al., 2017; Larroude et al., 2017); erythritol (Carly et al., 2017); mannitol (Rakicka et al., 2016); and various proteins (Celińska et al., 2018; Dulermo et al., 2017; Madzak, 2015) and organic acids (Markham et al., 2018; Blazeck et al., 2015). *Y. lipolytica* has also been engineered to produced aroma molecules such as 2-phenylethanol (rose-like odor; Celińska et al., 2013) and γ-decalactone (peach-like odor; Braga and Belo, 2016). In addition, we have witnessed the successful development of strategies that decrease bioprocess cost, namely by allowing *Y. lipolytica* to grow on cheap substrates, such as glycerol, sucrose, starch, inulin, cellobiose, or other waste products available for reuse (Cui et al., 2011; Johnravindar et al., 2018; Lazar et al., 2013; Ledesma-Amaro et al., 2015; Ledesma-Amaro and Nicaud, 2016a; Magdouli et al., 2017; Mirończuk et al., 2016; Spagnuolo et al., 2018; Yang et al., 2015). Strategies have also been developed to facilitate product extraction (Ledesma-Amaro et al., 2016a).

In this review, we describe and assess the most important synthetic biology tools developed to date for *Y. lipolytica*. We focus specifically on DNA assembly techniques, DNA parts for constructing expression cassettes, genome-editing techniques, and computational tools, and we discuss their potential to enhance this yeast's capabilities.

## 2 DNA assembly techniques

As the cornerstone of synthetic biology, the DNA assembly process allows the construction of novel biological systems and devices using defined, standardized, and well-characterized components. It is a procedure by which multiple DNA fragments are physically linked end to end, creating a target higher-order assembly that is then joined to a vector.

Traditional techniques employing restriction digestion and element-by-element cloning are time consuming and cost inefficient (Celińska and Grajek, 2013; Matthäus et al., 2014). Consequently, significant efforts are being made to develop better cloning strategies and DNA assembly techniques that would allow multigene cassettes to be constructed more quickly and efficiently. It would then be easier to build strains with complex genetic functionalities. These new techniques are also helping to increase the viability and/or transformability of recombinant strains, traits that are often impaired after several rounds of transformation.

**Fig. 1:**
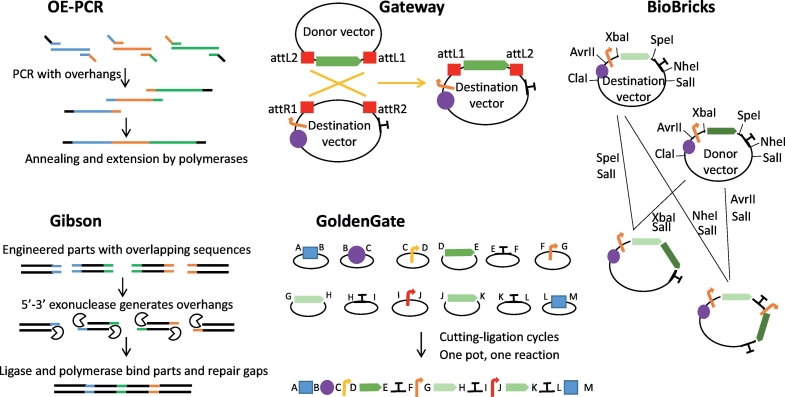
Summary of DNA assembly techniques. OE-PCR is a two-step PCR. During the first step, complementary overlapping overhangs are added to the parts to be assembled. During the second step, the parts hybridize with each other and form the new assembly via extension. In the Gateway method, the gene of interest, which has been cloned into the entry vector, is transferred into the destination vector via att site recombination. The expression vector obtained is then digested to release the expression cassette and used to transform Y. lipolytica. The BioBricks technique is used to clone parts via restriction digestion and the subsequent ligation of the resultant compatible sticky ends. YaliBricks vectors were designed to have AvrII, XbaI, SpeI, and NheI endonuclease site recognition. The ligation of the compatible overhangs produces a scar that is no longer recognized by either enzyme, which allows for subsequent assembly steps using more DNA parts. In Gibson assembly, parts are synthesized to overlap by 30+ bp. Their ends are then processed by an exonuclease that creates single-stranded 3′ overhangs, which facilitates annealing. The overhangs are fused together using a polymerase, which fills in gaps within each annealed fragment; a ligase seals gaps in the assembled DNA. Golden Gate assembly exploits type II enzymes, which cut outside their recognition sites to excise parts with arbitrarily defined four-base overhangs. Through the careful selection of compatible overhangs, such parts can be assembled altogether in a defined order. In the figure, the letters A to M represent different compatible 4-nt overhangs; the yellow, orange, and red arrows represent promoters; the green arrows represent genes; the violet circles represent markers; the blue squares represent insertion sequences; and the Ts represent terminators.

In this context, several well-known methodologies have recently been developed for *Y. lipolytica*. Here, we describe the most recent and relevant ones (Fig. 1).

### 2.1 One-step integration PCR

A simple and cost-effective method developed by Gao et al. (2014) allows the integration of multiple genes (four genes, total size of ~11 kb) by overlap extension PCR (OE-PCR). In their study, even though total efficiency was not very high (~15%), they were able to assemble β-carotene biosynthesis pathways rapidly, within a week's time, by dividing the pathways into four cassettes with ~50 bp overlaps between successive cassettes. The fragments were assembled into a single gene expression cassette that was then used to transform *Y. lipolytica* (Gao et al., 2014).

### 2.2 Gateway cloning

The Entry/Gateway® method employs site-specific recombination between att sites on interacting molecules to rapidly clone single DNA sequences in multiple destination plasmids (Hartley, 2000). It was recently adapted for use in *Y. lipolytica* by Leplat and colleagues. In their research, Gateway® vectors were combined, at the cloning site, with an overexpression cassette composed of the excisable *URA3* marker, the pTEF promoter, the LIP2 terminator, and zeta sequences, allowing random integration into the *Y. lipolytica* genome. As an example of the technique's utility, a library of alkaline extracellular protease (AEP) overexpression mutants was obtained in a single transformation experiment, using a novel high-throughput transformation method applied using 96-well plates (Leplat et al., 2015). This tool was used to construct more than 150 strains overexpressing individual transcription factors with a view to identifying regulators involved in lipid metabolism (Leplat et al., 2018).

### 2.3 BioBricks

Wong and colleagues developed a set of BioBrick-based vectors, called YaliBricks, for *Y. lipolytica*. It comprises four compatible restriction enzyme sites (*Avr*II, *Xba*I, *Spe*I, and *Nhe*I) that enable modular genetic engineering and the reuse of parts. Using this system, they were able to characterize 12 endogenous promoters and construct a five-gene biosynthetic pathway for producing violacein within a week's time (Wong et al., 2017). It is a fast and easy method, but it relies on specific restriction-site-free genes.

### 2.4 Gibson assembly

Gibson assembly allows multiple DNA fragments to be assembled regardless of their length or end compatibility and exploits three different enzymes: exonuclease, DNA polymerase, and DNA ligase (Gibson et al., 2009). Due to its ease of use and flexibility, it has rapidly been adopted as a DNA assembly method in a large range of microorganisms, including *Y. lipolytica*. For instance, Rodriguez and colleagues used the Gibson method to construct vectors to clarify the xylose pathway in *Y. lipolytica* (Rodriguez et al., 2016), while Bhutada and colleagues used it in research demonstrating that the deletion of glycogen synthase has beneficial effects on neutral lipid accumulation (Bhutada et al., 2017).

A similar method, which also uses homolog fragment ends and a single-tube enzymatic reaction, is employed in the commercial In-Fusion Kit (Clontech Laboratories, Inc). The In-Fusion enzymes also generate short regions of single-stranded overlaps between the DNA, facilitating directional assembly.

### 2.5 Golden gate

The Golden Gate (GG) modular cloning system utilizes type II restriction enzymes (Engler et al., 2008) and establishes a library of standardized and interchangeable DNA parts, which can subsequently be assembled in a single-step, one-pot reaction on a scaffold of predesigned 4 nt overhangs. Recently, a customized GG platform was developed for *Y. lipolytica* (Celińska et al., 2017). It can be used to express one-, two-, or three-customizable transcription units (TUs) in a versatile cassette comprising different genomic integration sites and recyclable auxotrophy markers. This fact means that thirteen elements can be assembled in a very fast and efficient manner. System viability and robustness was validated using a three-TU-bearing cassette encoding carotenoid synthesis genes (Celińska et al., 2017). One of the advantages of GG is that it allows combinatorial assembly, which can be used to efficiently generate libraries. Consequently, a promoter shuffling strategy using GG was used to screen optimum promoter-gene pairs for each transcriptional unit expressed. The best promoter combination was then used to engineer a lipid overproducer strain (Larroude et al., 2017); this research, through a combination of synthetic biology, metabolic engineering, and fed-batch fermentation, achieved the highest production level of β-carotene reported thus far, 6 g/L.

It is important to note that there are no perfect techniques for universal DNA assembly. Each method presents advantages and disadvantages in different situations. Therefore, method selection will depend on one's objectives.

OE-PCR and Gibson assembly are fast and simple, but specific primers are required for each assembly. As a result, these techniques are less versatile if, for example, the goal is to construct combinatorial assembly libraries. Moreover, as these methods are based on DNA annealing and polymerase elongation, it can be hard to correctly assemble hybrid promoters containing several copies of upstream activation sequences (UASs). Another constraint is that the final construct must be checked via sequencing to ensure there are no sequence errors due to polymerase amplification.

In the case of the BioBricks or GG techniques, it is necessary to construct a library encompassing the different parts to be used in the assembly. Library construction can be long, but, once it is completed, assembly is fast and versatile. There is also no need to verify the final assembly via sequencing (restriction-enzyme-based verification is enough). There are some important differences between the two techniques, however. Compared to GG, the BioBricks method requires to be devoided of greater number of restriction recognition sites. In addition, in GG, full assembly is carried out via a single reaction in a single pot. In contrast, when using BioBricks, parts are assembled one after the other, meaning that vectors should be digested, purified, and ligated at each cloning step. Finally, in GG, a reporter gene (e.g., red fluorescent protein) is used to preliminarily identify clones in which the assembly failed to be incorporated into the destination vector. As a result, GG is faster and easier to use.

The Gateway method does not involve high-throughput assembly technology, but it is very useful because the destination vectors of genes or constructions can be easily changed, which facilitates the functional analysis of genes and protein expression.

## 3 DNA parts for constructing expression cassettes

Controlling gene expression is a critical component of metabolic engineering, where the expression levels of the different enzymes in the pathway of interest must be balanced to maximize metabolic fluxes and minimize protein synthesis costs. Protein expression can be controlled by different means, including (but not limited to) transcription, mRNA stability, translation efficiency, or protein stability. Here, we discuss the most important tools that have been developed in *Y. lipolytica* to control the expression of genes, proteins, and other DNA parts that allow the creation of efficient integration cassettes and plasmids (Table 1).

**Table 1:** DNA parts used in expression cassette construction.

DNA Part	Characteristics	References
Promoters		
pTEF	constitutive; native	Muller et al., 1998
pTDH1, pGPM1, pFBA_IN_	constitutive; native	Hong et al., 2012
pEXP1, pGPAT, pGPD	constitutive; native	Xue et al., 2013
pGAP, pACL2	inducers were not determined; native	Wong et al., 2017
pXPR2	inducible by peptones; native	Ogrydziak and Scharf, 1982
pPOX2	inducible by fatty acids and alkanes; repressed by glucose and glycerol; native	Juretzek et al., 2000
pPOT1	inducible by fatty acids and alkanes; repressed by glucose and glycerol; native	Juretzek et al., 2000
pLIP2	inducible by fatty acids and alkanes; native	Sassi et al., 2016
pICL	inducible by ethanol, fatty acids, and alkanes; native	Juretzek et al., 2000
pYAT1	induced by nitrogen-limited conditions; native	Xue and Zhu, 2012
hp4d	hybrid promoter derived from pXPR2; contains four copies of UAS1XPR2 fused upstream from a minimal core LEU2 promoter; growth phase dependent; hybrid	Madzak et al., 2000
n UAS1_XPR2_-LEU	hybrid promoter derived from pXPR2; core minimal LEU2 promoter; n = number of UASs (up to 32); hybrid	Blazeck et al., 2011
n UAS1_XPR2_-TEF	hybrid promoter derived from pXPR2; core minimal TEF promoter; n = number of UASs (up to 16); hybrid	Blazeck et al., 2011
pEYK1, pEYD1	strongly induced by erythritol and erythrulose; repressed by glucose and glycerol; native and hybrid (p3AB-EYK)	Trassaert et al., 2017 Park et al., unpublished

Terminators		
XPRt	native terminator sequence	Franke et al., 1988
Lip2t	native terminator sequence	Pignede et al., 2000
Minimal XPRt	100-bp non-coding 3′ sequence	Swennen et al., 2002
CYC-t	*S. cerevisiae* terminator sequence	Blazeck et al., 2011; Mumberg et al., 1995
Synthetic	short synthetic terminators	Curran et al., 2015

Markers		
*LEU2, URA3, Lys5*	auxotrophy complementation	Barth and Gaillardin, 1996
*Ura3d4*	promoter-defective gene; several copies needed to restore auxotrophy	Le Dall et al., 1994
*SUC2*	gene from *S. cerevisiae*; sucrose utilization	Nicaud et al., 1989
*EYK1*	gene from *Y. lipolytica*; erythritol as carbon source	Vandermies et al., 2017
*ptxD*	gene from *Pseudomonas stutzeri*; growth in phosphite-containing media	Shaw et al., 2016
*Hph*	gene from *E. coli*; resistance to Hygromycin B	Otero et al., 1996; Tsakraklides et al., 2018
*nat1*	gene from *Streptomyces noursei*; resistance to nourseothricin	Kretzschmar et al., 2013; Tsakraklides et al., 2018
*guaB*	gene from *E. coli*; resistance to mycophenolic acid	Wagner et al., 2018
ylAHAS W572 L	*Y. lipolytica* mutant resistant to chlorimuron ethyl herbicide	Wagner et al., 2018

Tags		
tripeptide AKI or SKL	peroxisomal targeting signal	Xue et al., 2013
GPI anchor domains	signal for surface display; covalent bonds with cell wall β-1,6 glucans	Yue et al., 2008; Yuzbasheva et al., 2011; Moon et al., 2013
Flocculation domains	signals for surface display; non-covalent bonds with cell surface mannan chains	Yang et al., 2009
CBM	signals for surface display; non-covalent bonds with chitin	Duquesne et al., 2014
Pir	signals for surface display; covalent bonds with β-1,3 glucans	Duquesne et al., 2014
Oleosin C-t domain	targets lipid bodies	Han et al., 2013
Synthetic consensus secretory signal	MKFSAALLTAALA(S:V)AAAAA	Celińska et al., 2018
Fluorescent tag	reveals expression and localization	Bredeweg et al., 2017

Elements for exogenous DNA maintenance		
1 kb homologous flanking fragments	required for DNA integration by HR	Barth and Gaillardin, 1996
ZETA elements	integration at a zeta locus in zeta-containing strains; random integration in zeta-free strains	Pignede et al., 2000
rDNA	repeated genomic sequences	Le Dall et al., 1994
ARS68/ARS18	for maintenance of autonomously replicating vectors	Matsuoka et al., 1993; Fournier et al., 1993

GPI: glycosyl phosphatidyl inositol, CBM: chitin-binding module, Pir: protein internal repeat.

### 3.1 Promoters

In eukaryotic synthetic biology, selecting promoters according to their strength is the most widespread method for controlling gene expression in metabolic engineering techniques. However, it is important to note that higher protein expression is not always obtained using stronger promoters (Dulermo et al., 2017). Therefore, studies exploring promoter characteristics and engineering are needed to be able to consistently modify gene expression, which is a key parameter to be optimized in pathway engineering. Consequently, significant efforts have been made to develop promoters exhibiting a wide range of transcriptional activities.

In *Y. lipolytica*, the first strong promoters to be isolated and characterized were 1) the promoter from the *XPR2* (pXPR2) gene, which codes for an alkaline extracellular protease (Ogrydziak and Scharf, 1982), and 2) the constitutive promoter of *TEF* (pTEF), which codes for translation elongation factor-1 (Müller et al., 1998). Other native promoters that have been characterized or used to express heterologous genes in *Y. lipolytica* are pTDH1, pGPM1 (Hong et al., 2012), pEXP1, pFBAINm, pGPAT, pGPD, and pYAT (Xue et al., 2013). As mentioned earlier, Wong et al. (2017) selected and characterized eleven endogenous promoters, primarily associated with lipogenic pathways, in addition to the well-described pTEF promoter. pTEF was the most active promoter, followed by pGAP, pICL, and pACL2. The other promoters tested were pDGA1, pACC, pIDH2, pFAS2, pFAS1, pPOX4, pZWF1, and pIDP2.

To increase the current strength of available promoters, hybrid promoters have been developed. The functional dissection of pXPR allowed one of its UASs (UAS1_XPR2_) to be identified. Madzak and colleagues developed hybrid promoters containing up to four copies of UAS1_XPR2_ fused upstream from a core minimal LEU2 promoter, which were named the hp1d, hp2d, hp3d, and hp4d promoters, respectively (Madzak et al., 2000). These four strong hybrid chimera promoters resulted in a linear increase in promoter strength as a function of the number of tandem UAS1_XPR2_ elements (Madzak et al., 2000). The hp4d promoter is a widely used tool for heterologous gene expression in *Y. lipolytica*.

Since then, other hybrid promoters have been developed using this basic approach of associating multiple UAS tandem elements with a core promoter. Hybrid promoters containing up to 32 copies of UAS1_XPR2_ upstream from the minimal LEU2 core promoter and up to 16 copies of UAS1_XPR2_ upstream from the TEF core promoter have also been constructed. Some can increase expression efficiency eight-fold, compared to the known endogenous promoter in *Y. lipolytica* (Blazeck et al., 2011).

A general strategy for efficiently building synthetic promoters de novo is to both increase native expression capacity and to produce libraries for customizing gene expression; such approaches have been described by several groups (Blazeck et al., 2013, 2011; Dulermo et al., 2017; Shabbir Hussain et al., 2016; Trassaert et al., 2017). They identified novel UAS and core promoters, via promoter truncation and fragment dissection analysis, and created libraries of characterized strong hybrid promoters by fusing activating regions and core regions that were treated as independent synthetic parts. They identified UAS elements in the TEF promoter as well as the corresponding core minimal TEF promoter and then demonstrated that the ability of a UAS element to amplify expression is independent of the core promoter element. However, the magnitude of the amplification does depend on the core. Thus, the choice of both elements helps determine hybrid promoter strength, implying that it would be possible to design hybrid promoters displaying specific expression strengths. In other recent research (Shabbir Hussain et al., 2016), promoter strength was investigated by shuffling promoter constitutive elements (UAS, proximal promoter, TATA box, and core promoter); the results showed that gene expression can be fine-tuned by engineering such elements.

Another strategy used to enhance expression levels involves retaining an upstream intron with its corresponding promoter. Even though this intron-mediated enhancement (IME) has been observed in several organisms, including both plants and mammals, the mechanism remains elusive and is not effective for all introns (Gallegos and Rose, 2015). In *Y. lipolytica*, 15% of genes contain an intron (Neuvéglise et al., 2011), which means IME may play an important role in regulating gene expression in this organism. Hong and colleagues used pFBA with the native FBA intron (FBA1_IN_) and found that its strength was five times that of pFBA on its own. The efficacy of IME was further confirmed when a chimeric promoter, GPM1::FBA1, containing the 5′-region of the *FBA1* gene was attached to pGPM1 (Hong et al., 2012). The results of this study were similar to those previously described by Juretzek and colleagues, who discovered that expression levels climbed two-fold when pG3P contained the intron of *G3P* (pG3PB2), as compared to a promoter without an associated intron (Juretzek et al., 2000). The TEF_IN_ promoter has also been shown to increase gene expression levels 17-fold, compared to the constitutive TEF promoter (Tai and Stephanopoulos, 2013).

Inducible promoter systems offer the advantage of being able to control gene expression levels based on the presence of specific inducer or repressor molecules. This feature is desirable, for example, in fermentation processes because it allows the uncoupling of growth and production phases. The first of such promoters to be described were induced by hydrophobic substrates such as alkanes and lipids. These promoters are mainly encoded by the peroxisomal acyl-CoA oxidase 2 (POX2) gene, the peroxisomal 3-ketoacyl-thiolase (POT1) gene, the extracellular lipase Lip2 (LIP2) gene, and the cytochrome P450 oxidase (ALK1) gene (Juretzek et al., 2000; Sassi et al., 2016).

Recently, Trassaert and colleagues (Trassaert et al., 2017) isolated, characterized, and modified the promoter of the *EYK1* gene (pEYK1), which is induced by erythritol and erythrulose and repressed by glucose and glycerol. They showed that a hybrid promoter containing two additional tandem copies of the short (48-bp) UAS1_EYK1_ located upstream from the EYK1 promoter resulted in a 3.3-fold increase in expression. Deletion of the *EYK1* gene further improved expression levels: by preventing the catabolism of erythritol and erythrulose, induction was enhanced.

They are no complete report on the comparison of all available promoters. However, Darvishi et al. (2018) recently compared the relative strength of different promoters. We do know that promoters vary in size. Generally, native promoters range between 700 and 1000 bp in length, while synthetic promoters can exceed 1000 bp, depending on the number of repetitions they contain. That said, the recently described pEYK that includes regulatory elements useful in gene expression is only 300 bp long (Trassaert et al., 2017). The identification of *cis*-regulatory modules (CRMs) can help construct very short promoters, which can reduce assembly size and diminish the likelihood of homologous recombination due to repeated sequences.

### 3.2 Terminators

Terminators are essential for completing the transcription process because they affect mRNA stability and half-life and thus influence net protein synthesis (Geisberg et al., 2014; Mischo and Proudfoot, 2013). Native *S. cerevisiae* terminators have been successfully used not only in *Y. lipolytica* but also in other yeasts, suggesting a high degree of transferability across species (Wagner and Alper, 2016). Curran and colleagues recently evaluated a subset of short synthetic *S. cerevisiae* terminators in *Y. lipolytica* and found that GFP fluorescence was 60% greater, compared to results obtained using the wild-type CYC1 terminator. However, mRNA output was lower than in *S. cerevisiae*, indicating that there may still be some undefined organism-specific factors involved in termination (Curran et al., 2015).

Despite their importance, terminators have been studied in less detail than promoters, both in yeasts in general and in *Y. lipolytica* in particular. Creating de novo synthetic terminators would have benefits, such as minimizing the risk of undesirable homologous recombination. Also, shorter sequences could be used that have the same net effects as native terminators. Terminator studies are a promising area of research in *Y. lipolytica*.

### 3.3 Tags (secretion, localisation, and visualisation)

Different kinds of tags can be attached to homologous or heterologous proteins to direct them into different cellular or extracellular spaces, which is helpful for purification purposes or to compartmentalize pathway reactions. Some tags can also be used to determine protein localization or expression and thus partially or fully characterize a specific pathway. Here, we describe the tags most commonly used in *Y. lipolytica*.

Heterologous proteins can be tagged for 1) release into the cultivation medium, 2) display on the cell surface, or 3) incorporation into target intracellular organelles; fusion with a proper targeting sequence is required. For efficient secretion, a signal sequence is fused upstream from the mature sequence of the protein of interest. The most commonly used secretion signals are derived from the *Y. lipolytica XPR2* gene (which encodes the extracellulare protease AEP) or *LIP2* genes (which encode the extracellular lipase Lip2). However, other heterologous signal peptides (SPs) have also been used successfully (see reviews by Madzak, 2015; Madzak and Beckerich, 2013). Several anchor signals leading to surface display have also been developed, such as the glycosylphosphatidylinositol (GPI) anchor domain, which creates covalent bonds with cell wall β-1,6 glucans (Moon et al., 2013; Yue et al., 2008; Yuzbasheva et al., 2011); flocculation domains, which non-covalently bind to cell-surface mannan chains (Yang et al., 2009); chitin-binding modules (CBMs), which non-covalently bind to chitin in the cell wall (Duquesne et al., 2014), and the protein-internal-repeat (Pir) domain, which covalently binds to β-1,3 glucans (Duquesne et al., 2014). These anchor signals can be used in a variety of applications, such as bioconversion, biosensing, or high-throughput screening of enzymatic activity. Among the intracellular organelles, *Y. lipolytica* peroxisomes can be targeted by the peroxisomal targeting signal (PTS) domain, as the tripeptides AKI or SKL can be fused to the C-terminus of the protein (Haddouche et al., 2010; Xue et al., 2013). *Y. lipolytica* lipid bodies can be targeted by fusing the protein to the C-terminal domain of oleosin, a structural protein embedded in the phospholipid membrane of plant oleosomes (Han et al., 2013). The nucleus can be targeted by fusing the well-known viral SV40 nuclear localization sequence (NLS) (PKKKRKV) to the protein (e.g., for efficiently targeting Cas9; Schwartz et al., 2016). Other putative nuclear localization signals (Campos-Góngora et al., 2013) as well as putative mitochondrial targeting sequences (Bakkaiova et al., 2014; Kerscher et al., 2004) have been identified for *Y. lipolytica* proteins. However, no further analyses were done, to our knowledge, to characterize these endogenous targeting sequences. Systematic research focused on the identification and characterization of these endogenous targeting sequences is lacking.

Very recently, Celińska et al. (2018) analyzed the potential of 10 different SPs to facilitate the secretion of two heterologous proteins in *Y. lipolytica*. The study examined both previously described and novel SPs. The latter were identified via genomic DNA data mining and are native secretory proteins that are highly expressed in *Y. lipolytica*. Secretory capacity was assessed experimentally and compared with that obtained with known secretory tags. The most potent SPs turned out to be the novel SP1, SP3, and SP4 (from proteins encoded by YALI0B03564g, YALI0E22374g, and YALI0D06039g, respectively). The researchers also suggested a consensus sequence (MKFSAALLTAALA(S:V)AAAAA) for a potentially robust synthetic SP, which could be used to expand the molecular toolbox for engineering *Y. lipolytica*.

Using fluorescent proteins for characterizing cellular localization and expression is a well-known and widely used technique. In *Y. lipolytica*, this technique has been used to study and characterize transcription factors (Martinez-Vazquez et al., 2013) and hybrid promoters (Blazeck et al., 2013, 2011; Dulermo et al., 2017; Shabbir Hussain et al., 2016; Trassaert et al., 2017). A multipurpose vector for rapidly expressing fluorescently tagged proteins in *Y. lipolytica* was recently developed, which has streamlined analysis of protein localization (Bredeweg et al., 2017). In the latter study, the authors described the localization of enzymes involved in lipid synthesis. They also generated an atlas of strains with green fluorescent organelles, by tagging genes with GFP at their endogenous locus. These intracellular markers respond to native promoter controls and are non-essential proteins displaying consistently high levels of expression. This tool may facilitate cell biology research on organelles and on the colocalization of biosynthetic enzymes and pathways. Using green fluorescent protein (GFP) to tag organelle-specific proteins can overcome many of the limitations associated with dyes such as DAPI, Nile Red, or ER tracers, which include toxicity, poor penetration, and variability due to growth conditions, age, or nutrient availability (Bredeweg et al., 2017).

### 3.4 Plasmid vectors and genomic integration cassettes

In *Y. lipolytica*, like in other yeasts, the transforming vectors are shuttle vectors-hybrids between yeast- and bacteria-derived sequences. The bacterial component consists of a replication origin and a bacterial marker gene from *E. coli.* For expression in *Y. lipolytica,* a selection marker, transcriptional unit, and maintenance elements are needed. Two types of shuttle vectors, differing in their mode of maintenance in yeast cells, can be used: (i) episomal vectors (replicative) and (ii) integrative vectors (designed to be integrated into the yeast chromosome). As no natural episome has ever been detected in *Y. lipolytica*, replicative plasmids have been designed using chromosomal autonomously replicating sequence/centromere (ARS/CEN) replication origins (Fournier et al., 1993; Matsuoka et al., 1993). However, for the purposes of heterologous expression and/or genetic engineering, the use of such vectors is limited in *Y. lipolytica* because of low copy numbers (~1–3 plasmids/cell) and the high frequency of loss. That said, a two- to six-fold increase in gene expression was obtained using an expression cassette cloned into a replicative vector, as compared to the same cassette integrated into the genome (Nicaud et al., 1991). Replicative plasmids are of great interest for transient protein expression, like when Cre recombinase is produced for marker excision (Fickers et al., 2003) and Cas9 is produced for genome editing (Schwartz et al., 2016). Liu et al. (2014) engineered a replicative vector by fusing a promoter upstream from the CEN element. Although an 80% increase in plasmid copy number resulted, biased plasmid segregation was also observed.

Consequently, integrative vectors remain the vectors of choice (Madzak et al., 2000; Nicaud et al., 2002) for two main reasons. First, they are extremely stable. Second, they can be used to carry out multiple integration, with its correlated increase in gene expression. Multicopy integration can be achieved in *Y. lipolytica* by targeting repeated sequences (see below) or by using a defective marker, which allows more than 30 copies to be incorporated into the genome (Juretzek et al., 2001; Nicaud et al., 2002).

Integration into the genome can be achieved at specific target sites via homologous recombination. The process requires large (0.5–1 kb) and homologous 5′ and 3′ flanking regions. Integration can also take place within repeated genomic sequences, such as the rDNA region (Juretzek et al., 2001; Le Dall et al., 1994), and the long terminal repeated sequence of *Y. lipolytica*‘s retrotransposon, Ylt1, called the zeta region (714 bp) (Bordes et al., 2007; Juretzek et al., 2001; Nicaud, 2012; Pignede et al., 2000). Within the *Y. lipolytica* genome, around 200 copies of rDNA sequences are present; they are located on every chromosome (Casaregola et al., 1997) and have been used for multiple gene integration (Bulani et al., 2012; Celinska et al., 2016; Juretzek et al., 2001; Le Dall et al., 1994). Interestingly, when the zeta sequences on both sides of the integration cassette are used, insertion mainly occurs at a zeta locus in zeta-containing strains, while insertion is random in non-zeta-containing strains (Pignede et al., 2000). By taking advantage of the absence of Ylt1 in some strains, a specific locus integration platform has been developed that uses the zeta sequence to perform targeted integration at the *ura3* locus via a single cross-over event and homologous recombination (Bordes et al., 2007; Juretzek et al., 2001). Holkenbrink et al. (2018) identified 11 intergenic sites to be targeted using specific integrative expression vectors. These loci were selected with a view to promoting high gene expression levels and limiting the effects of expression cassette integration on growth.

To preclude the presence of bacterial DNA in the yeast strain after transformation, which can be a serious drawback, especially in commercial applications, “auto-cloning” expression vectors were developed (Pignede et al., 2000), from which the bacterial moiety can be removed prior to transformation by restriction digestion and agarose gel electrophoresis. The purified yeast expression cassettes are generally composed of an auxotrophy marker, the gene of interest under a specific promoter and terminator, and sequences for targeted integration into the genome; they can be used by themselves to transform the recipient strain.

### 3.5 Selection markers

Both genome integration and plasmid maintenance modification rely on selection markers. In *Y. lipolytica*, both auxotrophy and dominant markers are available. Auxotrophy markers, which can only be used with specific strains, remain the best choice for performing selection in *Y. lipolytica* (e.g., leucine, uracil, lysine, or adenine) (Barth and Gaillardin, 1996). A particular auxotrophy marker of note is *ura3d4*, a promoter-defective version of the *URA3* marker that is unable to correct auxotrophy when present in a single copy; thus, as mentioned before, it can be used to achieve multiple integration (Le Dall et al., 1994).

Dominant markers tend to be more broadly employed. Those that are available for *Y. lipolytica* include the *E. coli hph* gene (conferring hygromycin resistance) (Otero et al., 1996), the *Streptomyces noursei nat1* gene (conferring nourseothricin resistance) (Kretzschmar et al., 2013), the *Y. lipolytica* AHAS W572 L mutant (conferring chlorimuron ethyl herbicide resistance), the *E. coli guaB* gene (conferring mycophenolic acid resistance) (Wagner et al., 2018), and the *Streptoalloteichus hindustanus ble* gene (conferring zeocin resistance) (Tsakraklides et al., 2018). Other dominant markers involve the utilization of a specific carbon source, such as sucrose in the case of *S. cerevisiae* invertase expression (*SUC2* gene) (Nicaud et al., 1989a, Nicaud et al., 1989b, Lazar et al., 2013) or erythritol in the case of erythrulose kinase expression (*EYK1* gene) in a *Y. lipolytica* strain lacking this gene (Vandermies et al., 2017). However, use of these markers has proven to be difficult because of residual growth on sucrose impurities and the high level of spontaneous resistance in transformed cells, respectively (Barth and Gaillardin, 1996).

Shaw et al. (2016) engineered *Y. lipolytica* to exploit a naturally rare compound, potassium phosphite, and then turn around to supply phosphorus in a phosphate-deficient medium. The expression of phosphite dehydrogenase (*ptxD* gene) from *Pseudomonas stutzeri* can be used as a marker since it allows *Y. lipolytica* to grow in a phosphite-containing medium, which wild-type strains cannot do. The use of such rare compounds as source of nutrients can also prevent the growth of undesirable and/or foreign organisms. This competitive advantage can be of key importance in preventing contamination in large-scale fermentation operations; with minimal costs and the spread of antibiotic resistance genes is limited.

When creating strains harboring several integration cassettes, marker availability can be limiting. To solve this problem, Fickers and colleagues designed an approach combining the sticky-end polymerase chain reaction (SEP) method and the Cre-lox recombination system to facilitate efficient marker rescue and reuse. Upon expression of Cre recombinase, the marker was excised at a frequency of 98%, via recombination between the two lox sites. This method was shown to be very helpful in carrying out multiple gene deletions over a short period of time in *Y. lipolytica* (Fickers et al., 2003).

## 4 Genome-editing techniques

**Fig. 2:**
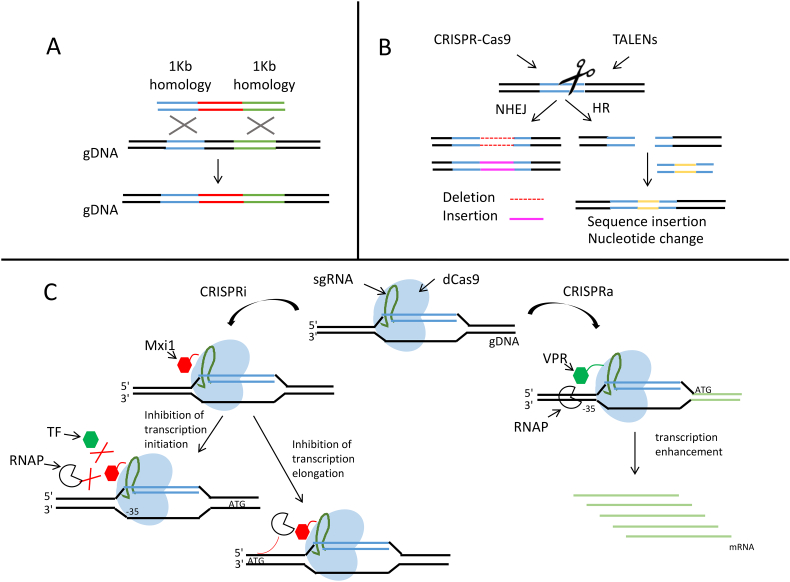
Chromosome editing tools and targeted genome engineering. A. Representation of the homologous recombination (HR) approach, which requires long (~1 kb) homologous flanking fragments to be efficient in *Y. lipolytica*. B. TALENs and Cas9 are programmable nucleases that recognize and bind to specific DNA sequences, causing double-strand breaks (DSBs), which induce non-homologous end joining (NHEJ) or HR. NHEJ introduces random insertions and deletions into the genome. Templates with homology arms can be added to take advantage of natural HR mechanisms to either modify single nucleotides or to insert new sequences. It should be noted that Cas9 introduces blunt breaks, while Fok1, the TALEN endonuclease, introduces a staggered cut (for simplicity, this difference is not shown in the figure). C. On the left, a CRISPR interference (CRISPRi) system is illustrated. The dCas9-sgRNA complex can either target the promoter inhibiting transcription initiation or target the gene sequence to prevent transcription elongation. On the right, a CRISPR activation (CRISPRa) system is illustrated. dCas9 is fused with a transcription factor and targets the upstream region of the gene, delivering the transcription factor to the promoter; this process enhances transcription efficiency. The abbreviations are as follows: gDNA: genomic DNA; sgRNA: single-guide RNA; dCas9: catalytically inactive Cas9; RNAP: RNA polymerase; TF: transcription factor; Mxi1: repressor; and VPR: synthetic activator domain (Schwartz et al., 2018).

Industrial-scale fermentation processes require that *Y. lipolytica* strains display a high degree of genetic stability; this stability is attained by incorporating genetic modifications into the chromosomes. When carrying out DNA repair, *Y. lipolytica* preferentially uses non-homologous end joining (NHEJ), as opposed to homologous recombination (HR) (Richard et al., 2005). This fact explains why long (~1 kb) homologous flanking fragments are required for HR (Fig. 2.a); however, their frequency of proper integration remains low. The *ku70* gene encodes a DNA-binding protein responsible for double-strand break repair during NHEJ. Its disruption significantly hinders NHEJ efficiency and increases the use of HR (Kretzschmar et al., 2013; Verbeke et al., 2013). Therefore, Δ*ku70* strains are commonly used for targeted gene insertion in *Y. lipolytica*.

As mentioned before, Fickers et al. (2003) designed a knock-out system for performing multiple gene deletions over a short period of time in *Y. lipolytica*. This process was achieved by constructing disruption cassettes harboring 1) regions homologous to the promoter and gene terminator regions intended for deletion; 2) an excisable marker with lox sequences on either side; and 3) a Cre recombinase that facilitates efficient marker rescue and reuse. Disruption cassette construction was later improved by using asymmetric *Sfi*I sites instead of *I-Sce*I, which simplified and speeded up assembly of cassette elements (Vandermies et al., 2017).

More recently, engineered nucleases that cleave specific DNA sequences in vivo have been developed for targeted mutagenesis. These nucleases enable efficient and precise genetic modifications to be carried out by inducing DNA double-stranded breaks (DSBs), which trigger DNA repair mechanisms that ultimately result in endogenous gene editing. The most widely used enzymes across different microorganisms are zinc-finger nucleases (ZFNs), transcription activator-like effector nucleases (TALENs), and Cas9 (Gaj et al., 2013); the latter two were recently developed for use in *Y. lipolytica*.

TALENs were created by fusing transcription activator-like effectors (TALEs) to the catalytic domain of the *Fok*I endonuclease. By customizing the TALE DNA binding domain, the DNA DSBs can be directed to occur at a specific target site (Christian et al., 2010) (Fig. 2.b). This technique was recently applied in *Y. lipolytica* to generate fatty acid synthase (FAS) mutants and proved to be very efficient in inducing targeted genome modifications: mutants were generated via error-prone NHEJ repair at the targeted locus in 97% of transformants. When homologous exogenous DNA was added to the TALEN targeted site, HR-mediated repair occurred in 40% of clones. This technique was used to directly produce site-directed mutagenesis in the *Y. lipolytica* genome (Rigouin et al., 2017).

A clustered regularly interspaced short palindromic repeats (CRISPR) technique can also be used. The CRISPR-Cas9 system consists of a Cas9-targeted nuclease that can be programmed with guide RNA (gRNA) to generate DSBs at specific DNA sites (Jinek et al., 2012) (Fig. 2.b). Recently, the CRISPR-Cas9 system from *Streptococcus pyogenes* was adapted to perform marker-free gene disruption and integration in *Y. lipolytica* (Schwartz et al., 2016). Schwartz and colleagues expressed the gRNA under a synthetic RNAP III promoter, and Cas9 was codon optimized for *Y. lipolytica*. Single-gene disruption and HR were more than 90% and 70% effective, respectively, when Cas9 and the gRNA were cotransformed using donor DNA. HR efficiency reached 100% when NHEJ was disrupted in the strain (Schwartz et al., 2016). Schwartz and colleagues also managed to integrate multiple genes at different loci without resorting to marker recovery. Nevertheless, gene integration efficiency depends on the integration site: of the 17 loci tested, 5 had high CRISPR-Cas9-mediated integration frequencies (48–62%) (Schwartz et al., 2017b). Around the same time, a second strategy for CRISPR-Cas9 genome editing in *Y. lipolytica* was developed (Gao et al., 2016). It involves expressing a human-codon-optimized Cas9 variant and gRNA flanked by ribozymes under the control of a RNAP II promoter; efficiency was 86% after four days of outgrowth. Both systems allow highly effective gene targeting.

Two other CRISPR tools have been developed for use in *Y. lipolytica*. Holkenbrink and colleagues created a toolbox, EasyCloneYALI, for easily performing genome editing in *Y. lipolytica* via CRISPR-cas9 technology—the standardized promoters, genes, and plasmids can be reused and easily exchanged (Holkenbrink et al., 2018). The researchers constructed a set of plasmids for integrating expression cassettes at a defined genomic locus; users can employ different selection markers or for the marker-free mode. Ku70p had been deleted in this study, making HR more efficient, and Cas9 was integrated into the genome and constitutively expressed. Eleven intergenic sites with high gene expression levels were identified, but only five had efficiencies higher than 80% for marker-free integration. Very recently, Gao et al. (2018) developed a dual CRISPR-cas9 strategy using paired gRNA to create complete gene knockout via gene excision. Basically, two vectors, each containing a Cas9 gene and a single-guide RNA (sgRNA) cassette, are cotransformed in *Y. lipolytica*. The gRNAs were designed to target areas upstream from the start codon and downstream from the stop codon, which led to complete gene excision when the breaks occurred simultaneously and the resulting genomic regions were end-joined. The strategy was tested on six genes, and excision efficiency reached about 20%. The researchers also used this dual CRISPR-cas9 strategy to incorporate donor DNA into the excision region using marker-free integration (i.e., the integrated cassette had no selection marker). Then, a single vector containing the Cas9 gene and the two sgRNAs was constructed, and cotransformation occurred with another vector containing the donor cassette. Integration efficiency ranged from 15% to 37%, depending on the method (HR or homology-mediated end-joining, respectively).

A CRISPR technique was also developed for controlling gene expression. Schwartz and colleagues adapted CRISPR interference and activation (CRISPRi and CRISPRa) systems for use in *Y. lipolytica*. In these systems, a catalytically inactive Cas9 (dCas9), which is able to bind to DNA complementary to the gRNA spacer sequence but unable to introduce DSBs, targets the promoter region of a gene of interest, repressing or activating transcription, respectively (Fig. 2.c). First, the researchers showed that the system functioned for gene repression (Schwartz et al., 2017a)—for 8 of the 9 genes tested, at least 50% of transcription was repressed using a multiplex strategy. Repression was enhanced when the Mxi1 repressor, but not the KRAB repressor, was fused with dCas9. Finally, the repression of *KU70* and *KU80* led to an HR efficiency of 90%. Later, this same group developed a CRISPRa system to activate genes in *Y. lipolytica* (Schwartz et al., 2018). They screened four different activation domains and several target sites in the promoter region. By adding the VPR activation domain to dCas9 and choosing gRNA targeting locations upstream from the core promoter, they activated two native β-glucosidases genes, *BglI* and *BglII*, which allowed growth on cellobiose. Indeed, Guo and colleagues engineered a *Y*. *lipolytica* strain capable of degrading cellobiose, thanks to the overexpression of these two endogenous genes (Guo et al., 2015).

Zhang et al. (2018) used a CRISPRi system for repressing genes in *Y. lipolytica*—four different repressors (dCpf1, dCas9, dCpf1-KRAB, and dCas9-KRAB) were employed. As it was difficult to achieve strong repression levels with a single gRNA element and identify effective target sites, the group exploited a multiplex gRNA strategy. Gene repression efficiency exceeded 80% when the *gfp* gene was targeted at three different sites. As shown by Schwartz et al. (2017a), the KRAB domain does not influence dCas9 activity; however, compared to results for dCpf1 alone, the use of dCpf1-KRAB increased repression efficiency by about 30%.

The development of the TALEN and CRISPR-Cas9 systems was an important step in modern genomic engineering. Due to their simplicity, efficiency, and affordability, they have both become key genome-editing tools. However, the CRISPR-cas9 system has some advantages: compared to the TALEN system, its molecular tool is much easier to produce, and the technique is also suitable for multiplex genome editing. That said, the fact that TALENs cause breaks only upon dimerization of the *Fok*I domain increases system specificity and reduces the risk of off-target effects. Regarding the use of the CRISPR-Cas9 system to carry out multiple disruptions, progress must still be made in *Y. lipolytica* to reach efficiency levels comparable to those in *S. cerevisiae*, which were 65% for a sextuple gene deletion (Mans et al., 2015) and 96% for a quadruple deletion (Ferreira et al., 2018). Recently, a CRISPR-Cpf1 system was shown to be very efficient for simultaneous gene disruption in *S. cerevisiae*: efficiency levels reached 88% and 100% for a quadruple deletion (genomic integration of Cpf1 vs. Cpf1 expression from a multicopy plasmid, respectively; Swiat et al., 2017). With further development, these strategies could serve as helpful tools for increasing the efficacy of multitarget mutations in *Y. lipolytica*.

## 5 Host strains

*Y. lipolytica* displays interstrain variability: different strains have different morphologies and metabolite patterns as they grow (Egermeler et al., 2017); they may also have genomic differences (Naumova et al., 1993). As previously underscored in the literature, in metabolic engineering, the choice of the parental strain is key to optimum system performance (Abghari et al., 2017; Larroude et al., 2017; Steensels et al., 2014; Xie et al., 2015). A summary of the most commonly used strains is presented below and in Table 2.

**Table 2:** Most commonly used *Yarrowia lipolytica* strains.

Strain	Genotype and characteristics	References
E150	*MATb* his-1 *leu2–270, ura3–302, xpr2–322, pXPR2-SUC2* reference strain, genome sequenced, grown on sucrose, Leu−, Ura−	Barth and Gaillardin, 1996
W29	*MATa*, French wild-type strain sequenced genome	
H222	*MATa*, German wild-type strain	
CBS6124	*MATa*, American wild-type strain	
Po1d	*MATa*, *leu2–270, ura3–302, xpr2–322, pXPR2-SUC2* derived from W29, extracellular protease AEP deleted, grown on sucrose, Leu−, Ura−	Le Dall et al., 1994
Po1f	*MATa*, *leu2–270, ura3–302, xpr2–322, axp1–2, pXPR2-SUC2* derived from Po1d, both extracellular proteases deleted, grown on sucrose, Leu−, Ura−	Madzak et al., 2000
Po1g	*MATa*, *leu2–270, ura3–302::URA3, xpr2–322, axp1–2, pXPR2-SUC2* derived from Po1f, both extracellular proteases deleted, grown on sucrose, pBR docking platfrom, Leu−	Madzak et al., 2000
Po1h	*MATa*, *ura3–302, xpr2–322, axp1–2*, *pXPR2-SUC2* derived from Po1f, both extracellular proteases deleted, grown on sucrose, Ura−	Madzak, 2003
Y1212	*MATa*, *leu2–270, ura3–302, xpr2–322, lip2Δ, lip7Δ, lip8Δ, Leu2-Zeta, pXPR2-SUC2* derived from Po1d, zeta platform, lipΔ, the three main lipases deleted, Ura−	Bordes et al., 2007

The most widely used *Y. lipolytica* genetic backgrounds are the wild-type French strain W29 (CLIB89), the wild-type German strain H222 (DSM 27185), the wild-type American strain CBS6124–2, and the wild-type Polish strain A101 (Barth and Gaillardin, 1996; Wojtatowicz et al., 1991). The reference strain for *Y. lipolytica* is E150 (CLIB122), whose genome has been fully sequenced and annotated (Dujon et al., 2004). This strain was derived from multiple back-crosses between the W29 and CBS6124–2 strains (Barth and Gaillardin, 1996). The genome sequence of W29 is now also available (Magnan et al., 2016). Genetic and physiological differences are naturally present among the strains. First, in contrast to the European strains, the American strain has a retrotransposon dispersed throughout its genome, Ylt1, which can also occur as solo zeta elements (Ylt1's long terminal repeats) (Barth and Gaillardin, 1996; Schmid-Berger et al., 1994). As mentioned above, zeta elements function as HR sites, and, due to their high copy number in the genome, they can be used as a target for the multiple integration of exogenous DNA (Juretzek et al., 2001; Pignede et al., 2000). Second, H222 naturally overproduces α-ketoglutarate, which is used in industrial processes as a building block for synthesizing heterocycles and also serves as a dietary supplement (Yovkova et al., 2014). Additionally, strains can differ in their filamentation profiles (Barth and Gaillardin, 1997; Thevenieau et al., 2009). The hyphal form can be 100% undesirable, causing fermenter clogs or low production yields, or 100% necessary, such as in immobilized cell bioreactors (Vandermies et al., 2018).

The most commonly used recipient strains are Po1 series strains (Po1d, f, g, and h), which were derived from the wild-type strain W29 (Le Dall et al., 1994; Madzak et al., 2000). They have been engineered to express the heterologous gene *SUC2* from *S. cerevisiae*, which allows sucrose to be used as a carbon source. This trait is of particular interest in industrial applications because it means yeast can exploit molasses, a cheap and abundant agroindustrial substrate (Nicaud et al., 1989a, Nicaud et al., 1989b). Both extracellular proteases (AEP and AXP) have been deleted from Po1f, g, and h, making them more suitable for heterologous protein expression (Madzak et al., 2000). Furthermore, Po1d and H222 derivatives, in which *Ku70* and/or *Ku80* were deleted, were constructed to increase HR efficiency (Kretzschmar et al., 2013; Verbeke et al., 2013).

Some derivative strains have also been built for more specific applications. The Y1212 strain, derived from Po1d, is equipped with an integrated zeta docking platform, for facilitating the incorporation of zeta-based integrative vectors. The three main lipase-encoding genes (*LIP2*, *LIP7* and *LIP8*) have been deleted in this strain, which makes it suitable for genetically engineering lipid metabolic pathways (Bordes et al., 2007). This strain was developed to be able to efficiently compare activity levels among enzymes or variants. Activity can be quantified directly in the supernatant, rendering protein purification and quantification unnecessary. This is possible because of expression level reproducibility, attributable to the integration of a single copy of the expression cassette at the zeta docking platform. Therefore, activity patterns are due to enzymatic differences at the molecular level and not to differences in expression levels (Cambon et al., 2010).

Other *Y. lipolytica* chassy strains of interest include strains for producing humanized glycoproteins, which help bypass the immunogenic problems associated with recombinant therapeutic proteins (De Pourcq et al., 2012a, De Pourcq et al., 2012b) or strains for bioconverting cellulose, an abundant and renewable carbon source, into products of commercial interest (Guo et al., 2018). Strains have also been engineered to take advantage of a greater substrate range, allowing cheaper substrates to be used as carbon sources with a view to reducing fermentation costs. These strains were recently reviewed by Ledesma-Amaro and Nicaud (2016a).

It is clear that *Y. lipolytica* strains display a broad range of features, and several options are available when selecting the appropriate host strain. However, other strains of interest remain to be developed, such as strains with a disrupted *EYK1* gene, which would allow the use of the inducible promotor pEYK (described above), or strains resistant to high concentrations of glucose and glycerol.

It would also be interesting to better characterize the physiological features of the *Y. lipolytica* wild-types and to evaluate their natural traits as they relate to such biological processes as filamentation, intermediate metabolite production (e.g., of citric acid, succinate, alpha-ketoglutarate, and erythritol), lipid production, and resistance to antibiotics. As shown by Egermeier et al. (2017), environmental conditions can also have an impact. The researchers examined the ability of different strains to convert glycerol into polyols and citric acid under two different pH conditions; significant differences in metabolite patterns were observed. This finding shows that metabolism is dependent on the environment, a discovery that was also reported by Tomaszewska et al. (2014, 2012). This knowledge helps clarify *Y. lipolytica*'s physiology and the underlying regulatory mechanisms, information that will inform strain choice, which will depend on the desired application. As new synthetic biology techniques, genome-editing tools, and expression cassette insertion methods become available, it will become quicker and easier to transfer metabolically engineered modifications into other wild-type strains.

## 6 Computational tools

The advent of whole-genome sequencing and the reconstruction of metabolic networks at the genome scale have enabled the development of computer-assisted design tools that can be used to guide metabolic engineering. Such tools are important for building novel biosynthetic pathways and improving fluxes in existing pathways. More specifically, these models can be used to predict the outcomes of genetic modifications and metabolic responses to environmental conditions as well as to determine optimal engineering strategies (Fernández-Castané et al., 2014; Oberhardt et al., 2009). The construction of accurate genome-scale metabolic models (GEMs) is crucial for properly simulating cell behavior, which requires high-quality annotated genome sequences and experimental data (Aung et al., 2013). Five GEMs have been developed thus far for *Y. lipolytica* (Table 3), which we will describe below.

**Table 3:** **Comparison of the different GEMs available for *Y. lipolytica*.** The number of genes, reactions, and compartments were determined using the suite package sybil (Gelius-Dietrich et al., 2013). Accuracy values correspond to the values published by the authors. ND: not determined.

**Name**	**No. genes**	**No. reactions**	**No. compartments**	**Accuracy**	**References**
iNL895	898	1989	16	0.65	Loira et al., 2012
iYL619_PCP	619	1142	2	0.83	Pan and Hua, 2012
iMK735	735	1337	8	0.8	Kavšček et al., 2015
iYali4	901	1985	16	ND	Kerkhoven et al., 2016
iYLI647	646	1343	8	ND	Mishra et al., 2018

*Y. lipolytica* was fully sequenced in 2004, as part of the Génolevures program, and the quality of manual annotation is high (Dujon et al., 2004; Sherman et al., 2009). The first functional GEMs were built by Loria and colleagues in 2012 (Loira et al., 2012), by combining in silico tools and manual curation; using a *S. cerevisiae* model as a scaffold; and validating efforts using previously published experimental data. For the *Y. lipolytica* iNL895 model, there was a fair degree of concordance between the model's growth predictions and the experimental results (accuracy: 0.65).

At almost the same time, another GEM was developed by Pan and Hua (Pan and Hua, 2012). In this case, the metabolic network, iY619_PCP, was reconstructed using genome annotation and information from biochemical databases such as KEGG, ENZYME, and BIGG. The in silico model successfully predicted growth in minimal media and on different substrates (accuracy: 0.83). The authors also used flux balance analysis (FBA) with single-gene knockouts to predict gene essentiality. Flux variability analysis (FVA) was employed to design new mutant strains that redirect fluxes toward lipid production.

Another GEM model for *Y. lipolytica*, named iMK735, was created based on a *S. cerevisiae* model (iND750) by Kavšček et al. (2015). The model was manually curated for species-specific reactions, and then FBA was used to design fermentation strategies where lipid production was optimized. Concordance between model predictions and experimental results was high (accuracy: 0.80). In addition, the model correctly predicted that a reduced aeration rate would induce lipid accumulation.

A fourth GEM model, iYali4 (Kerkhoven et al., 2016), was constructed using the recently described Yeast 7.11 consensus network (Aung et al., 2013) and curated to include unique reactions from both iYL619_PCP and iNL895. It was used to study the regulation of lipid metabolism. The researchers carried out integrative analysis of multilevel omics data obtained from *Y. lipolytica* chemostat cultures grown under carbon- and nitrogen-limited conditions. They showed that the previously documented increase in lipid accumulation after nitrogen depletion was not regulated at the transcriptional level but, instead, was related to amino-acid metabolism.

Very recently, Mishra and colleagues built a new objective-oriented model for simulating long-chain dicarboxylic acid (DCA) production. This new GEM, named iYLI647, was constructed using the iMK735 model as a scaffold, and manual curation was performed to expand the model's characteristics. For example, reactions from the ω–oxidation and the β –oxidation pathways were incorporated; the model also allowed the separation of biomass synthesis equations for growth under carbon- and nitrogen-limited conditions, enhancing the accuracy of growth predictions relative to previous models. The model was then used to identify genetic engineering targets with DCA overproduction in mind (Mishra et al., 2018).

Traditional GEMs have limitations as they incorporate only stoichiometric constraints; they assume steady-state metabolite concentrations. Thus, efforts are being made to integrate kinetic information into the models. Several dynamic mathematical models have been developed to help optimize lipid contents and/or to study citric acid production in batch and continuous processes (Arzumanov et al., 2000; Papanikolaou and Aggelis, 2003a, Papanikolaou and Aggelis, 2003b; Papanikolaou et al., 2006). However, these models still do not consider internal regulation of metabolism and metabolic shifts.

Recently, Robles-Rodriguez et al. (2017) came up with three dynamic metabolic models, based on a simplified metabolic network, for describing lipid accumulation and citric acid production by *Y. lipolytica* growing on glucose. These models can guide the design of strategies for improving culture performance via the identification of rate-controlling steps and metabolic fluxes. Three independent experimental data sets, obtained from fed-batch and sequential-batch cultures of *Y. lipolytica* grown on glucose under conditions of nitrogen limitation and deficiency, were used to calibrate and validate the models. The models' predictions were reasonably close to the experimental data. A common advantage of these dynamic metabolic models is that they can incorporate metabolic descriptions and regulation mechanisms and can thus be used to identify rate-controlling steps in the metabolic network.

At a higher scale, high-throughput technologies make it possible to analyze large amounts of omics data, facilitating the investigation of cell metabolism and physiology at the systems level. In recent years, such research has been carried out in *Y. lipolytica*. For instance, transcriptome analysis revealed the existence of four different transcription profiles over a 32-h fermentation period and identified genes potentially involved in the metabolism of oleaginous species (Morin et al., 2011). A separate transcriptome analysis, carried out in tandem with proteomics methods, was used to explore amino acid catabolism (Mansour et al., 2009; Morín et al., 2007). Additionally, proteome analyses were conducted in *Y. lipolytica* to characterize the proteins involved in the yeast-to-hypha transition (Morín et al., 2007); the osmotic response to erythritol (Yang et al., 2015); and the degradation of TNT (Khilyas et al., 2017). Fluxomics has grown as a discipline thanks to 13C-based metabolic flux analysis. The latter was used to discover that the pentose phosphate pathway is the major source of the cofactor required for lipid production (Wasylenko et al., 2015). The response to nitrogen limitation, and its effects on lipid storage regulation, was analyzed by Pomraning et al. (2016) using a multiomics approach. Another multiomics study found that carbon fluxes were redirected from amino acids to lipids in *Y. lipolytica* grown in carbon- and nitrogen-limited chemostat cultures (Kerkhoven et al., 2016). In subsequent work, the same researchers showed that leucine biosynthesis was particularly downregulated when the yeast was grown under nitrogen-limited conditions, concomitantly with lipid accumulation (Kerkhoven et al., 2017). A recent study looked at transcriptional changes in *Y. lipolytica* during lipid biosynthesis in strain carrying a *MHY1* gene inactivation (Wang et al., 2018); *MHY1* encodes a C_2_H_2_-type zinc finger protein. They found that nearly 25% of annotated *Y. lipolytica* genes were expressed at significantly different levels, suggesting Mhyp plays a crucial regulatory role in various biological processes, including lipid and amino acid metabolism (again underscoring the interaction between these pathways). Trebulle and colleagues (2017) used transcriptomic data gathered during lipid accumulation to infer the gene regulatory network; the goal was to identify regulators involved in lipid accumulation. The nine highest ranked transcription factors were then overexpressed in a wild-type strain over the course of a systematic high-throughput functional analysis carried out by Leplat et al. (2018); overall, 148 putative transcription factors were overexpressed in this study. For six of the nine mutants obtained, lipid content was at least 10% greater than that in the wild-type, which validates the utility of the GRN approach for identifying context-specific transcription factors.

Genome-scale modeling contributes significantly to our understanding of cellular processes and is very useful for guiding metabolic engineering via the improvement of strain performance. However, since there are differences in GEM curation procedures and coverage of metabolites and reactions differs, the models vary in prediction accuracy. The optimal model will depend on one's objectives. That said, efforts should continue to improve the models and their predictions. For example, additional components, such as omics data and/or enzyme kinetics and abundance, could be included in GEMs, as it has been recently done for *S. cerevisiae* (Sánchez et al., 2017).

## 7 Conclusions and perspectives

In this review, we have attempted to describe the state-of-the-art synthetic biology tools available for *Y. lipolytica*, a micro-organism of industrial importance. We also discussed the most commonly used DNA parts and genetic engineering techniques used with this yeast. Some of these methods have the distinct potential to become standard lab techniques for *Y. lipolytica*. The greatest promise is held by tools and methods for identifying and characterizing new parts, carrying out DNA assembly, and performing genome editing.

It is important to note that many more synthetic biology tools are available for the model organisms *S. cerevisiae* and *E. coli* because they are well characterized, grow quickly, and are easy to stably transform. However, other yeasts are often more desirable as bioprocessing hosts because their natural metabolisms render them more suitable for producing the target product. With the development of new synthetic biology tools, some previously non-conventional yeasts, such as *Y. lipolytica* or *Pichia pastoris*, are on their way to becoming model organisms (Gellissen et al., 2005; Löbs et al., 2017; Wagner and Alper, 2016). The impact of these tools is evidenced by the increase in the number of engineered strains. Other yeasts, like *Kluyveromyces marxianus* and *Rhodosporidium toruloides*, have certain features that make them suitable for industrial applications. However, their molecular tools are less well developed. This state is expected to change in the coming years as the development of synthetic biology tools continues (Lane and Morrissey, 2010; Park et al., 2018a, Park et al., 2018b).

*Y. lipolytica* is considered to be non-pathogenic and is generally regarded as safe. Its ability to generate large amounts of biomass on simple substrates makes it a good host for producing pharmaceutical compounds and food additives, among other products, especially given the availability of its fully sequenced genome and diverse metabolic engineering toolkit. The synthetic biology tools described in this review do not only serve as proof of concept; in many cases, they have already been used to engineer *Y. lipolytica* strains. Several recent reviews discuss the products and production yields obtained with these strains (Darvishi et al., 2018; Shabbir Hussain et al., 2016; Ledesma-Amaro and Nicaud, 2016b; Liu et al., 2015; Madzak, 2018, 2015; Markham and Alper, 2018; Xie, 2017), underscoring *Y. lipolytica*'s potential as a cell factory.

Synthetic biology is an emerging engineering discipline and, as such, applies key engineering concepts such as standardization, modularity, predictability, reliability, and modeling in its work with biological systems (Andrianantoandro et al., 2006).

Most of the standard promoters and other DNA parts used for engineering *Y. lipolytica* have not been studied rigorously enough to meet synthetic biology standards for predictability and reliability. For example, parts must be characterized under standardized conditions, and strain background must be taken into consideration. While efficient wild-type and hybrid promoters have been developed, such as the strong constitutive pTEF, the phase-dependent hp4d, the fatty-acid-inducible pPOX2, and the erythritol/erythrulose-inducible promoter pEYK1, more inducible promoters are needed so that gene expression can be switched on and off. In addition, it is crucial to confirm the reproducibility of the promoter-related results obtained by different labs.

Modularity in *Y. lipolytica* is also currently being explored (although it is still in an early stage of development), thanks to the recent creation of modular cloning systems such as the above-mentioned Golden Gate system (Celińska et al., 2017). It would be highly beneficial to add novel, fully characterized parts to this toolbox.

Models of *Y. lipolytica* metabolism keep increasing in number, and it is difficult for non-experts to evaluate which model is the most appropriate for a given application. This challenge has been partially dealt with in other organisms such as *S. cerevisiae*, where researchers joined together to create a consensus model (Herrgård et al., 2008). Such a strategy could be applied for *Y. lipolytica*: taking the best parts of existing models to create a baseline model that could be improved through refinement.

Although many additional advances are needed before *Y. lipolytica* can obtain the status of a model organism, significant efforts are being made so that this yeast can be fully exploited. Consequently, it is expected that new research will continue to improve the techniques describe herein and to develop innovative tools and technologies aimed at better engineering *Y. lipolytica* strains.
